# On the Causes of Insanity in the United States

**Published:** 1878-05

**Authors:** 


On the Causes of Insanity in the United States.—Insanity is on the increase in the United States, and has been for many years. It is but reasonable to suppose that the perverted business and industrial relations of the past four years have given it an additional impetus, although little appears in asylum records under this head. There are now some fifty thousand persons in the insane asylums of the United States (that is equal to twice the number of our regular army), and many others treated outside asylums. No man can declare himself absolutely safe from an attack of insanity, whether his family history be clear of it or not. It is no respecter of persons; it attacks high and low, learned and unlearned.

The most accurate statistics gleaned teach "that one person in every sixteen hundred and ninety of the population will become of unsound mind in the course of each and every year." If this ratio be correct, a population of

44,000,000 will annually add 26,035 persons to the list of the insane, not speaking of the numbers that will have accumulated in prior years. This annual yield, at a cost of keeping of $266.71 per capita (the average cost in thirty-six of our most cheaply conducted asylums), will cost the State—for the State stands in *loco parentis* to the afflicted—$6,956,398; this sum, added to the cost of sheltering, $52,-070,000, estimated at $2,000 per capita ($3,000 is the usual estimate), will impose on the State an annual burden of $59,026,000, or an average yearly tax (were it so levied) of $1.34 for every man, woman and child in the United States.

In view of the many-sided importance of this subject, it deserves and demands the studious attention of all men. As physicians, it becomes us to know all that is known of this "sorest of all maladies;" as philanthropists to prevent, and when we cannot prevent, alleviate; as intelligent citizens and tax-payers that we may know how to expend to the best advantage the millions that are annually expended for the maintenance of this numerous corps of the invalid army of the republic. What, then, constitutes insanity, and what are the chief causes that produce it? Waiving technicalities, that man or woman is insane, in the eye of the law, whose mental operations are so impaired by disease that the individual is no longer able to take care of himself (or herself) and of his (or her) estate. It simply means of unsound mind—of diseased brain. It has nothing to do with the supernatural—with spirits, demons, or even with Wilkie Collins' monsters; and the sooner this idea, which still hovers round as a relic of ages of ignorance and barbarism, is abolished, the better it will be for the afflicted ones. There is no sense in the peculiar odium which is associated with the insane state in the minds of many; it is a compound of ignorance, pride and ungodlinesss.

The principal forms of insanity are the hereditary and non-hereditary. To have hereditary insanity means that one's parents or more remote ancestor's were insane, or suffered from other nervous disease, which morbid taint appears in the offspring in the form of pure insanity; for all nervous diseases are interchangeable—that is to say, any nervous affection may in a subsequent generation appear as that same nervous affection, as some other nervous disease, or as insanity, unsound mind. Non-hereditary insanity is that which appears in an individual whose family history is free from insanity and other well-marked nervous diseases.

The greatest factor in the production of both forms of insanity, hereditary and non-hereditary, is over-indulgence in alcoholic liquors. It has recently been claimed that liquor, directly or indirectly, in one generation or another, is the cause of all insanity, but this is absurd. Disease attacks the brain as well as other bodily organs, from common causes incident to human life, and to the delicate mechanism of our cerebral machinery. It is a safe and moderate estimate to say that it causes one-half of all the madness that exists; it, therefore, muddles more than 13,000 brains, and damns more than 13,000 souls per annum, in this country alone. Is it not the duty of the State to protect its citizens? It should prohibit, by stringent laws, severely executed, the sale of those poisons sold as alcoholic beverages, and it should and ought to regulate the sale, even of pure liquors, both of which blast and pauperize so many people, and so largely increase the taxation.

Having thus mentioned what is generally admitted to be the greatest factor in the causation of insanity, it is not an easy matter to enumerate all the causes that produce it, in the order of frequency, for the reason that some cases are due to more than one cause. Paresis, which contributes some fifteen per cent. of the inmates of our private asylums (twenty per cent. to the English private asylums) is a disease of *wine and women.* Perhaps, however, it is safe to say, indeed my little experience warrants me in saying, that sexual excess is by far the more efficient element of this compound cause in the production of paresis. Sexual excess has been proven in every case of paresis I have ever seen, and at the present writing I have quite a number in my care. Paresis may result, in rare instances, from over-taxation of the mind in the direction of legitimate business. Sexual excess, with and without strong drink, produces unsoundness of mind in other forms than paresis, and thus swells the proportion of insanity caused by it much higher than that given for paresis. All estimates are but approximates. Self-abuse produces some ten per cent. The "wastes and burdens of life," losses, sorrows, griefs, disappointments, over-tension of the mind in the pursuit of good and laudable objects, especially over-draughts on the imagination, lead to mental aberration.—*Medical and Surgical Reporter.*

